# Frequency of extra pulmonary complications in critically ill COVID-19 patients and their association with inflammatory markers and hypoxia: Retrospective analysis at a tertiary care hospital in Karachi, Pakistan

**DOI:** 10.12669/pjms.39.6.7380

**Published:** 2023

**Authors:** Sadaf Hanif, Muhammad Sohaib, Syed Talha, Asma Rayani

**Affiliations:** 1 Sadaf hanif, FPCS COVID ICU Aga Khan University Hospital, Karachi, Pakistan; 2 Muhammad Sohaib, FCPS COVID ICU Aga Khan University Hospital, Karachi, Pakistan; 3 Syed Talha Sibtain, MBBS COVID ICU Aga Khan University Hospital, Karachi, Pakistan; 4 Asma Rayani MBBS COVID ICU Aga Khan University Hospital, Karachi, Pakistan

**Keywords:** ARDS, COVID-19, Extra pulmonary complications

## Abstract

**Background and Objective:**

This study aimed to determine the incidence of extra pulmonary complications among critically ill COVID-19 patients requiring invasive mechanical ventilation and association of these complications with various inflammatory markers and degree of hypoxia.

**Methods:**

A retrospective cohort study was conducted among 173 adults in Karachi having COVID-19 and were admitted to ICU in a tertiary care private hospital between August 2020 to July 2021.

**Results:**

The median age of patients included in the analysis was 61 years (IQR; 16). Acute kidney injury, septic shock, cardiac injury, and electrolytes imbalance were the most frequent extra pulmonary complications with proportion of 65.3% (n=113), 63.6% (n=110), 61.8% (n=107) and 33.5% (n=58). Statistically significant differences in the median serum levels of ferritin were observed among male versus female, critically ill covid patients with and without ICU mortality as well as patients with and without hospital mortality (p-value <0.05). Significantly higher serum levels of d-dimer were noted among patients who developed acute liver injury or NSTEMI, or had ICU stay of > 3 days or received mechanical ventilation for >2 days.

**Conclusion:**

Acute kidney injury, septic shock, cardiac injury, and electrolytes imbalance were the most common extra pulmonary complications among mechanically ventilated COVID-19 patients with ARDS. Higher serum d-dimer levels were associated with acute liver injury, NSTEMI, ICU stay longer >3 days and invasive mechanical ventilation >2 days. Higher serum ferritin levels are associated with male sex and serve as an important predictor of ICU as well as hospital mortality.

## INTRODUCTION

Global evidence suggests that besides serious pulmonary manifestations COVID-19 also manifests as various extra pulmonary complications as result of inflammatory process or direct organ damage.^1^ Acute liver injury, acute kidney injury, cardiac injury, neurological dysfunctions, and coagulopathy are among common extra pulmonary complication of COVID-19.^2^,^3^ However, the development of ARDS among COVID-19 patients also serves as an important predictor for the development of various extra pulmonary complications as well as mortality.^4^-^6^ A systematic review of 27 cohort studies and six case series involving 42,219 participants from variety of populations concluded that critically ill COVID-19 patients are at risk high mortality rates.^7^ The study also reported mortality rate of 48% among South Asian population excluding China. The study also supported the role of clinical interventions like invasive mechanical ventilation, renal replacement therapy and vasopressors in improving the patients` survival during ICU stay.^7^

A study conducted in 2019 among COVID-19 patients in Karachi identified acute liver injury and acute kidney injury as the most common extra pulmonary complications with frequency of 58.9% and 24.4% respectively. The study reported significantly higher mortality among COVID-19 patients with ARDS as compared to patients without ARDS. The study showed higher morbidity and mortality among patients with complications like cardiomyopathy, renal impairment, and shock.^8^ However, there is lack of scientific evidence regarding the role of inflammatory markers and degree of hypoxia in development of extra pulmonary complications among COVID-19 patients requiring invasive mechanical ventilation. So, this study was conducted with the purpose to determine the frequency of extra pulmonary complications and possible association of the inflammatory markers and hypoxia with extra pulmonary complications among critically ill COVID-19 patients. Our study will help in identifying common extra pulmonary complications among critically ill COVID-19 patients, as well as help in understanding the role of inflammation markers and hypoxia in occurrence of such complications. Such information can be useful in risk stratification among critically ill COVID-19 ICU patients with ARDS managed with invasive mechanical ventilation.

## METHODS

A Retrospective Cohort Study was conducted among COVID-19 patients admitted to ICU of a tertiary care hospital in Karachi from August 2020 to July 2021. Any critically ill COVID-19 patient with acute respiratory distress syndrome (ARDS) who was admitted to ICU and received invasive mechanical ventilation was included in the study. The proposal for this research study was reviewed by Ethics Review Committee (ERC) of Aga Khan University, Karachi. An ethical exemption letter was provided by the ERC as the study did not involve any direct interaction with human subjects (ERC Reg# 2022-7571-21939). In-total medical records of 173 COVID-ICU patients were included in the study. ARDS was diagnosed using Berlin Criteria defined as PaO2/FiO2 ratio of <300 at the time of ICU admission and bilateral radiographic pulmonary infiltrates.^9^ The data was extracted using a structured data collection form and analyzed using SPSS version-24.

Descriptive statistics were computed for demographic and health-related characteristics. Mann-Whitney-U test was applied to compare any statistically significant differences in the median levels of inflammatory markers and PaO2/FiO2 ratio based on demographic and health-related characteristics. P-value of 0.05 or less was considered statistically significant.

## RESULTS

The median age of 173 critically COVID-ICU patients included in this study was 61 years (IQR=16 years). 76.3% (n=132) of all the patients were male while 23.7% (n=41) were female. 94.2% (n=163) of all the patients were found to receive mechanical ventilation for more than two days of duration, (Table-I).

**Table-I:** Demographic and Clinical characteristics of critically ill COVID-19 patients admitted with ARDS in COVID-ICU of a private, tertiary careteaching Hospital in Karachi (n =173).

Median (IQR)	Frequency (n)	Percentage (%)
***61.0years (16years)***		
***Age***		
40 years and less	12	6.9
41 years -64 years	92	53.2
66 years and above	69	39.9
***Sex***		
Male	132	76.3
Female	41	23.7
***Burden of Co-morbids***		
No or One comorbid	79	45.7
Two or more co-morbids	94	54.3
***History of Co-morbids***		
Diabetes Mellitus	90	52.0
Ischemic Heart Disease	30	17.3
Hypertension	109	63.0
Chronic Kidney Disease	20	11.6
Chronic Liver Disease	05	2.9
COPD	11	6.4
***Hospital Mortality***		
ICU mortality	91	52.6
Non-ICU Mortality	13	7.5
No Mortality	69	39.9
Duration of ICU Stay *		
***Median (IQR)***		
≤3 days	11	6.4
>3 days	162	93.6
***Days on ventilator***		
***Median (IQR)***		
≤2 days	10	5.8
>2 days	163	94.2

**Fig.1:**
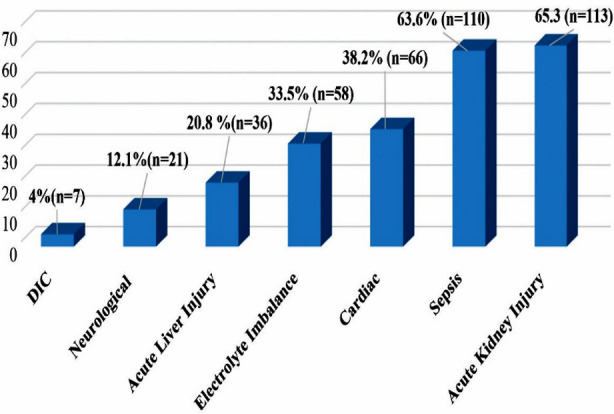
Incidence Proportion for various extrapulmonary complicationsn among critically ill COVID-19 patients admitted with ARDS in COVID-ICU of a private, tertiary care teaching Hospital in Karachi (n =173).

The study calculated the incidence proportion for all the extrapulmonary complications among critically ill COVID-19 patients. Acute kidney injury, sepsis, cardiac complications, electrolyte imbalance, and acute liver injury were identified as the most frequent extra-pulmonary complications followed by neurological complications and disseminated intravascular coagulation (DIC) (Fig.1).

Our study found no statistically significant differences in the median levels of inflammatory markers based on different age groups, status for medical comorbid, and duration of hospital stay. Nevertheless, this study found significantly higher levels of serum ferritin among male patients as compared to female patients (p-value <0.05). The study also found significantly higher serum ferritin levels among those who either had ICU or hospital mortality as compared to those who did not have ICU or hospital mortality (Table II). Similarly, significantly higher serum levels of d-dimer were observed among patients who had an ICU stay of > three days and those who received invasive mechanical ventilation for > two days (Table II).

**Table-II:** Difference in median levels of CRP, Ferritin, LDH and PaO2/FiO2 ratio across critically ill, COVID-19 patients on mechanical ventilation with different demographic and clinical characteristics (n =159).

Variable	Median (IQR)				

	CRP	Ferritin mcg/L	D-dimer mg/dl	LDH U/L	PaO2/FiO2 ratio
***Age***					
40 years and less	89(123)	988(1242)	3.5(5.1)	714(447)	168(135)
41 years -64 years	124(151)	1042(872)	2.5(4.0)	681(373)	118(89)
66 years and above	102(115)	1009(1329)	4.0(7.4)	583(298)	131(103)
***Sex***					
Male	110(135)	1178(989) *	3.1(5.9)	655(356.5)	130(103)
Female	105(161)	462(862)	2.8(5.1)	573(241.5)	112(92)
***Burden of Co-morbids***					
No or one comorbid	115(127.5)	1178(1089.3)	3.0(6.5)	609(347.3)	124103)
Two or more co-morbids	105(146)	924(1036.5)	3.1(5.3)	631(334.5)	130(97)
***Diabetes Mellitus***					
Yes	107(142)	966.0(1039)	3.0(4.3)	645(371.0)	122(91)
No	109(131.5)	1058(1086)	3.1(7.3)	602.5(313.5)	127.5(108)
***Ischemic Heart Disease***					
Yes	78.5(127)	935(1214)	3.3(7.1)	727(368)	116(89)
No	116(138)	1052(1031)	3.0(5.0)	623(309)	125(104)
***Hypertension***					
Yes	106(140)	924(1009)	3.0(5.7)	630(329)	121(92)
No	121(129)	1265.5(178.8)	2.9(5.2)	622.5(362)	135.5(108)
***Chronic Kidney Disease***					
Yes	144(127)	1286(1192)	3.6(4.7)	532(204)	124(138)
No	106(135)	966(1056)	3.0(5.7)	658(356)	124(96)
***Chronic Liver Disease***					
Yes	34(56)	1323(5732)	3.7(2.7)	550(160)	248(201)
No	110(138)	1009(1055)	3.0(5.7)	630(351)	124(97)
***COPD***					
Yes	167(182)	924(2398)	4.0(1.9)	719(492)	106(43)
No	106.5(135)	1040(1051)	3.0(5.6)	626(329)	127(106)
***Duration of ICU Stay***					
≤3 days	53(127)	675(1265)	1.0(2.2)	666(439)	124(188)
>3 days	110(139)	1040(1019)	3.2(6.3)*	625(326)	127(96)
***Days on ventilator***					
≤2 days	56(153)	554(838)	1.6(2.3)	647 (453)	124(112)
> 2 days	110(137)	1084(1062)	3.2(6.1)*	628(328)	125(99)
***Hospital Mortality***					
Yes	105(130)	1259(1159)*	3.4(7.0)	672(384)	130(100)
No	130(45)	810(785)	2.9(3.5)	614(289)	119(96)
***ICU Mortality***					
Yes	98(133)	1264(1136)*	3.4(7.2)	684(392)	122(94)
No	122(142)	803(913)	2.9(4.1)	605(279)	130(105)

-Kruskal Wallis test was applied for variables with more than two categories.

This study did not find any significant statistical differences in the serum levels of d-dimer among patients with or without extrapulmonary complication except for acute live injury. Likewise, significantly higher d-dimer levels were observed among patients who developed NSTEMI as compared to those with no NSTEMI (Table III).

**Table-III:** Difference in median levels of CRP, Ferritin, D-dimer, LDH and PaO2/FiO2 ratio across critically ill, COVID-19 patients on mechanical ventilation with and without extra pulmonary complications (n =159)

Variable	Median (IQR)				

	CRP	Ferritin mcg/L	D-dimer mg/dl	LDH U/L	PaO2/FiO2 ratio
***Sepsis***					
Yes	98(133)	979(1099)	3.2(6.6)	604(316)	134(101)
No	125(149)	1084(1047)	2.3(4.8)	685(429)	112(97)
***DIC***					
Yes	84(186)	1241(857)	1(3)	729(147)	150(150)
No	109(134)	1021(1068)	3.1(6.2)	625.5(347)	124(99)
***Acute Liver Injury***					
Yes	84(125)	1456.5(1705)	4.8(6.1) *	720.5(462)	106(97)
No	110(140)	924(1047)	2.8(5.0)	622(311)	130(104)
***Acute Kidney Injury***					
Yes	109(138)	1118(1277)	3.2(5.0)	605(390)	130(99)
No	110(138)	912(1044)	2.9(6.6)	655(282)	115(100)
***Neurological Complication***		753(1358)		685(318.5)	140(118)
Yes	110(130)	1040(1026)	2.0(5.4)	628(329)	124(97)
No	109(136)		3.1(5.6)		
***Cardiac Complication***					
Yes	106(133)	1032(1122.5)	3.1(5.5)	631(339)	131(95)
No	110(143)	1030.5(1097)	2.9(6.6)	613(337)	116.5(106)

* P value <0.05- statistically significant difference in the median levels of d-dimer.

The study didn’t find statistically significant differences in the degree of hypoxia measured as PaO2/FiO2 ratio among patients with and without extrapulmonary complications or based on differences in sociodemographic characteristics.

## DISCUSSION

This study was specifically conducted among critically ill COVID-19 patients who developed ARDS and received invasive mechanical. The demographic characteristics of COVID-19 patients included in the study were comparable to the previous evidence from Karachi.^8^ Moreover, acute kidney injury, sepsis, cardiac complications, and acute liver injury were identified as the most frequent extra-pulmonary complications. This finding is in line with previous international and local studies and explains the high mortality among critically ill COVID-19 patients in the local population.^10^-^14^ The local evidence also supports the considerably high burden of acute liver injury and acute kidney injury among COVID-19 patients with severe and prolonged disease.^15^,^16^

This study did not find any statistical differences in the serum levels for most of the inflammatory biomarkers i.e., CRP, LDH, ferritin, and d-dimer based on differences in age, sex, types, and number of previous medical comorbidity, ICU mortality, and hospital mortality. This can be explained by the previous local evidence where the rise in biomarkers among hypoxic COVID-19 patients was later followed by a decline and high mortality among patients with invasive ventilation.^12^ However, male patients were found to have significantly higher serum ferritin levels as compared to females. This finding is in line with the current evidence identifying the male sex as the major predictor of the worst prognosis and higher risk of mortality among COVID-19 patients.

Our study could not find any significant differences in the inflammatory markers among different age groups. This study estimated COVID-19-associated ICU mortality of 52.6% which is slightly higher than ICU mortality reported among COVID-19 patients from other parts of Asia except China.^7^ The relatively higher mortality in our study sample can be attributed to the inclusion of COVID-19 patients with severe ARDS which itself has been identified as an independent predictor of hospital or ICU mortality. 4-6

Significantly higher levels of serum ferritin were observed among patients with ICU and hospital mortality in comparison to patients without ICU and hospital mortality respectively.^15^ This finding is in line with the previous evidence identifying raised serum ferritin levels as another predictor for disease severity as well as hospital and ICU mortality among critically ill COVID-19 patients.^11^-^13^

We observed significantly higher serum levels of d-dimer among patients who developed acute liver injury as well as among those who developed Non-ST Elevation Myocardial Infraction (NSTEMI) as compared to those who didn’t develop the mentioned complications. Previous studies have been reporting a positive association of high levels of d-dimer with the development of kidney injury, liver injury, as well as COVID-19 associated mortality.^15^-^20^ However, previously published literature shows mixed evidence regarding the association of d-dimer levels with myocardial infarction and its severity among COVID-19 patients hence requires further research.^21^-^23^ This study didn’t observe any significant association between d-dimer and kidney injury which is contrary to the previous evidence; however, this can be explained by the differences in the study population as well as the severity of disease.^20^

In addition, patients who had ICU stay of > three days or received mechanical ventilation for > two days were also found to have significantly higher median levels of d-dimer than those patients with ICU stay of ≤ three or who received mechanical ventilation for ± two days. This finding is in line with previous evidence supporting the influence of high d-dimer levels at third day of hospital admission in predicting hospital mortality among COVID-19 patients.^17^,^24^ This finding is also supported by a study conducted by Tassiopoulos and colleagues which highlights the role of d-dimer-driven anticoagulation therapy in improving survival among intubated COVID-19 patients admitted to ICU.^25^

Nevertheless, our study could not find any significant difference in the proportion of extrapulmonary complications among COVID-19 patients with and without ARDS based on the PaO2/FiO2 ratio. This finding is in contrast to previous studies identifying low PaO2/FiO2 ratio (OR=0.96, as an independent risk factor of prolong hospital stay, disease severity as well as mortality among COVID-19 patients.^26^-^28^ However, our study findings can be well explained and supported due to the inclusion of critically ill COVID-19 patients only.

This study provides evidence regarding the frequency of extrapulmonary complications in the local context as well as about the role of inflammatory markers among critically ill COVID-19 patients with ARDS requiring invasive mechanical ventilation. The study identified ARDS as an independent predictor of ICU and hospital mortality among critically ill COVID-19 patients in the local context. Moreover, this study also determined the association between serum ferritin levels and ICU and hospital mortality and the association of d-dimer levels with acute liver injury and NSTEMI.

### Limitations:

It notably focused on critically ill COVID-19 patients with ARDS, receiving invasive mechanical ventilation which has affected the utility of its findings. Moreover, this study couldn’t find significant differences among most of the inflammatory markers among patients with and without extrapulmonary complications. This can be well explained by the high median age of the study sample, the limited sample size, and the specific inclusion of critically ill patients with ARDS. Furthermore, this study lacks the capacity to report extrapulmonary complications developed after hospital discharge.

## CONCLUSION

Acute kidney injury, septic shock, cardiac injury, and electrolyte imbalance were the most common extrapulmonary complications among mechanically ventilated COVID-ICU patients with ARDS. However, higher serum levels of d-dimer were associated with acute liver injury, NSTEMI, ICU stay > three days, and mechanical ventilation > two days. Higher serum ferritin levels were associated with male sex and serve as an important predictor of ICU as well as hospital mortality.

### Authors Contribution:

**SH:** Conceived the idea, proposal and Performa development, manuscript writing. She was the principal Investigator as well as responsible for the accuracy or integrity of the work.

**MS:** Manuscript writing and proof reading.

**ST** and **AR**: Data Collection.
